# Supplementary figures and images for: Glomerular endothelial derived vesicles mediate podocyte dysfunction: A potential role for miRNA
Source: PLoS One. 2020 Mar 26;15(3):e0224852. doi: 10.1371/journal.pone.0224852 (PMC7098579; doi:10.1371/journal.pone.0224852)

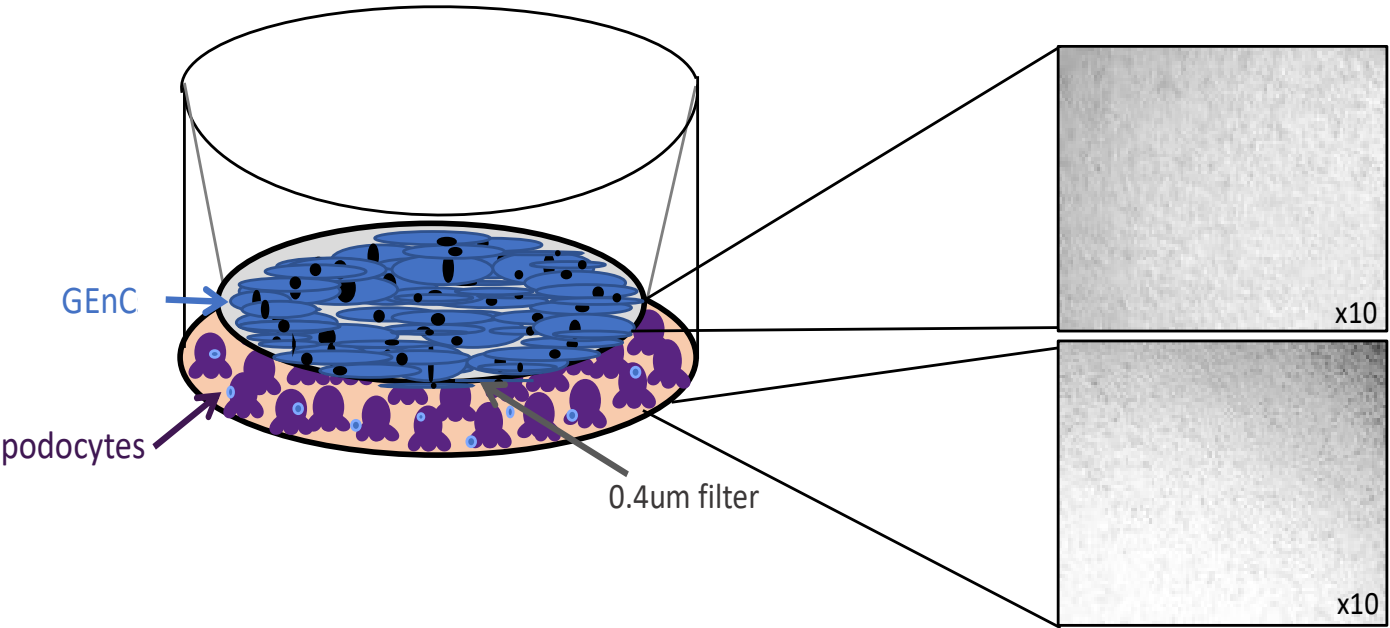

Supplement: S1 Fig — Illustration of the transwell culture of GEnC and podocytes, allowing both cell types to communicate through the transfer of EV. Cells are plated and cultured for 7 days. GEnC are seeded onto a 0.4μm filter. Podocytes are seeded onto the bottom of the well. (PDF) [file pone.0224852.s001.pdf]

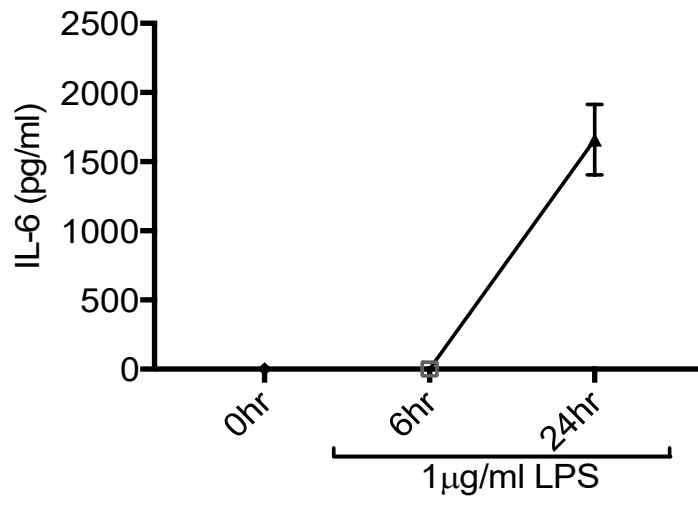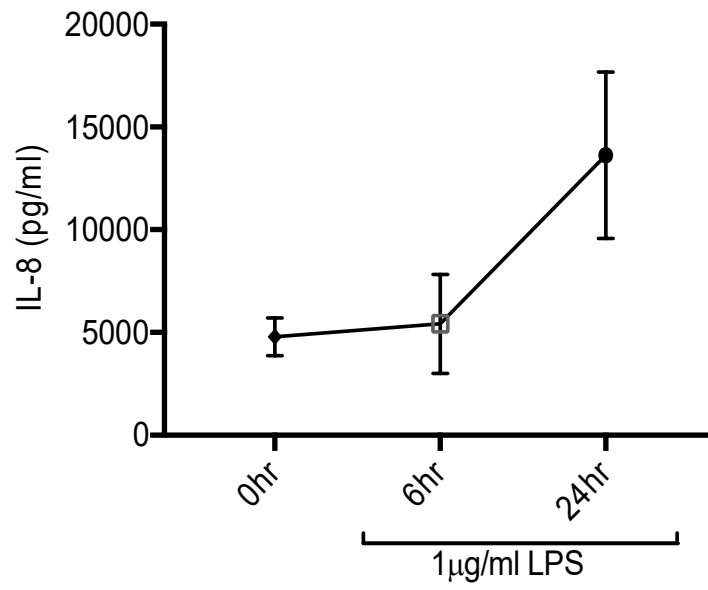

Supplement: S2 Fig — GEnC were treated with 1μg/ml LPS for 6 or 24 hours. Supernatants of LPS treated GEnC were collected. IL-6 and IL-8 secretion was measured by ELISA and compared to untreated GEnC. At least 4 readings of experiments were performed in duplicate. (PDF) [file pone.0224852.s002.pdf]

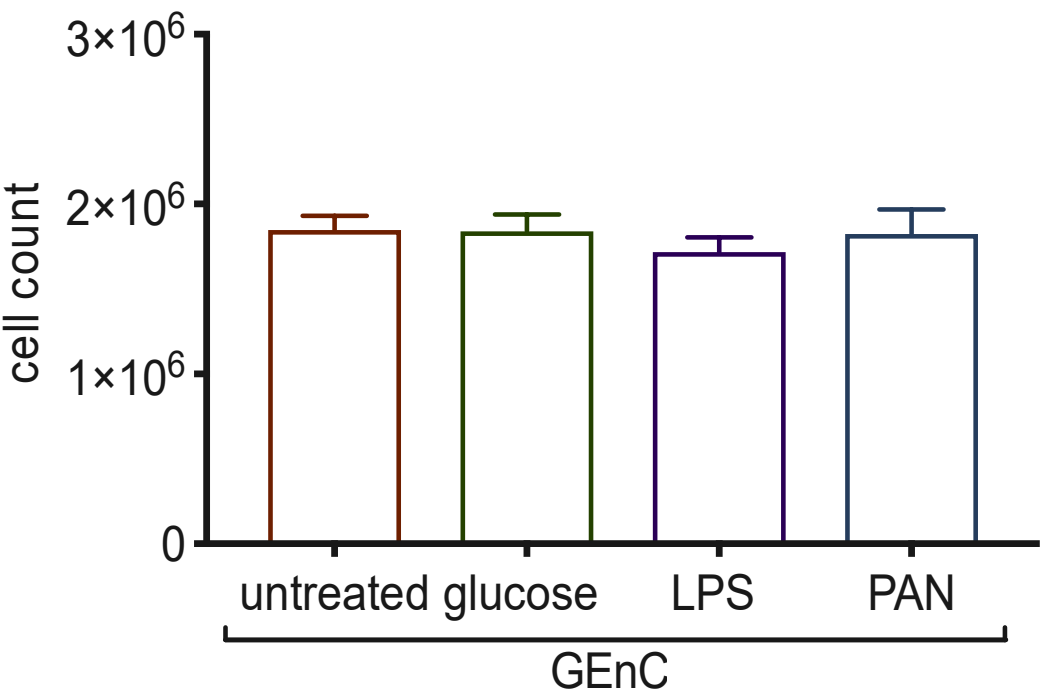

Supplement: S3 Fig — GEnC were left untreated or stimulated with 30 mmol glucose, 1ug/ml LPS, 100ug/ml PAN for 24hours. Cells were counted using a haemocytometer and compared to untreated cells for viability. n = 6. (PDF) [file pone.0224852.s003.pdf]

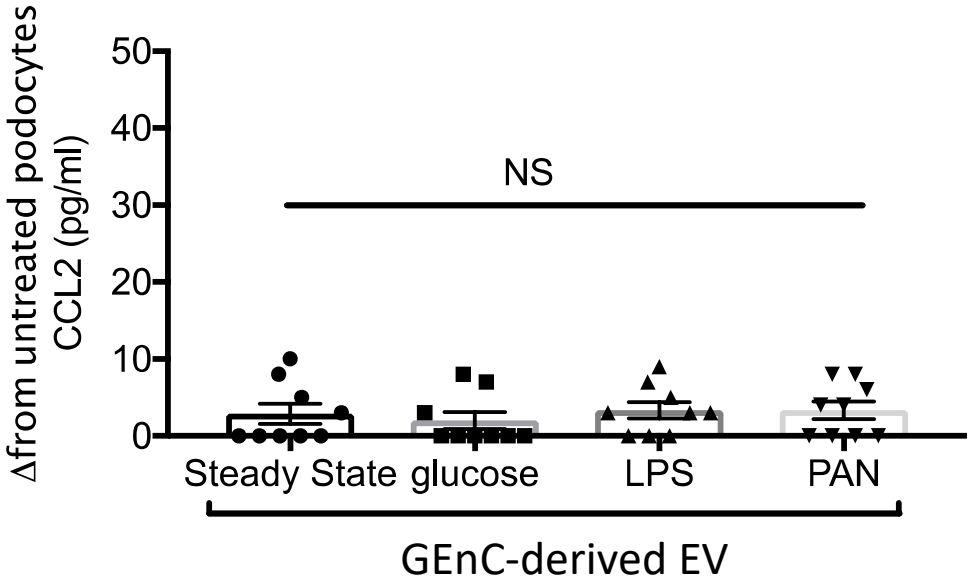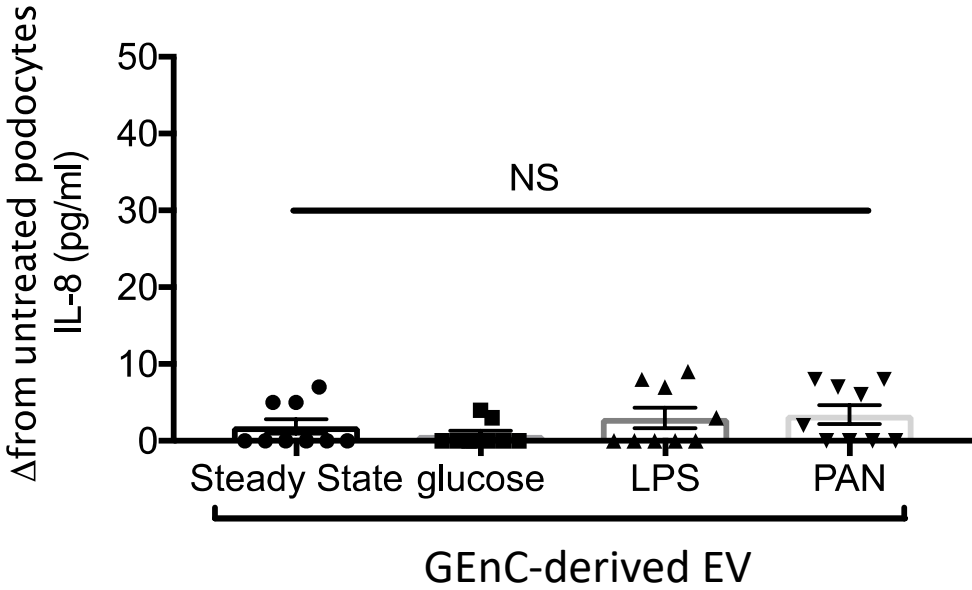

Supplement: S4 Fig — Podocyte supernatant was collected 24-hour post incubation with GEnC EV. Podocytes remained untreated or were incubated with EV from steady state, glucose, LPS or PAN treated GEnC. CCL2 and IL-8 expression was measured by ELISA. Data presented as change in expression from untreated podocytes. n = 7. (PDF) [file pone.0224852.s004.pdf]

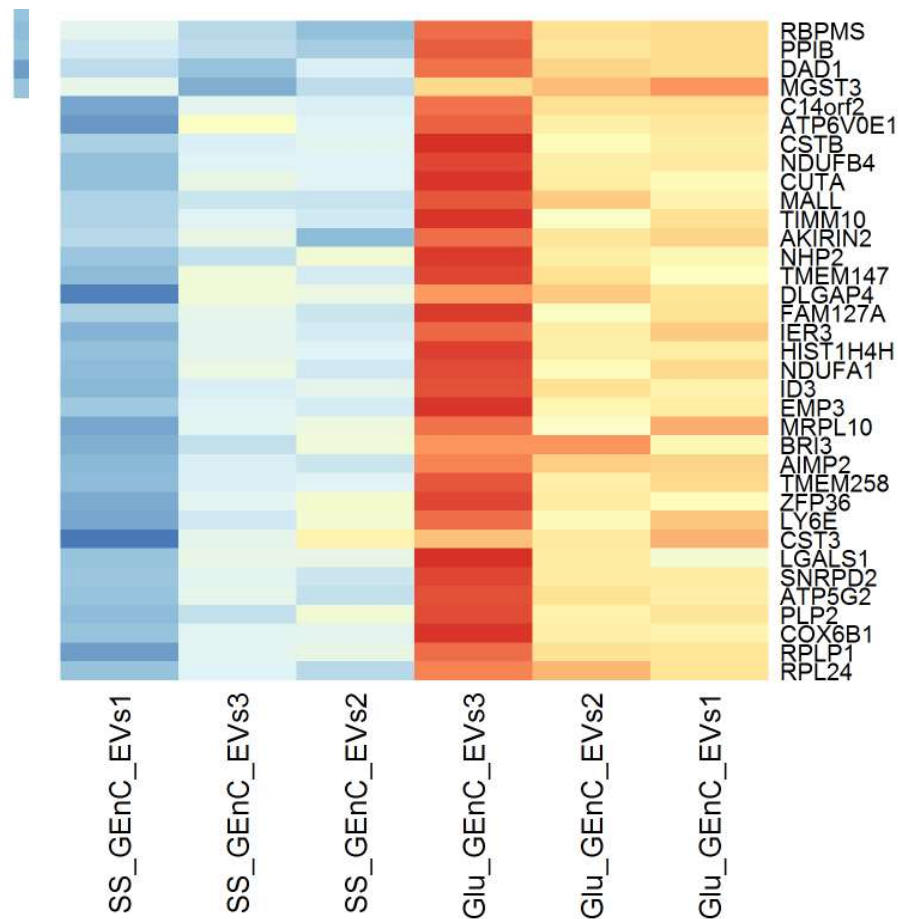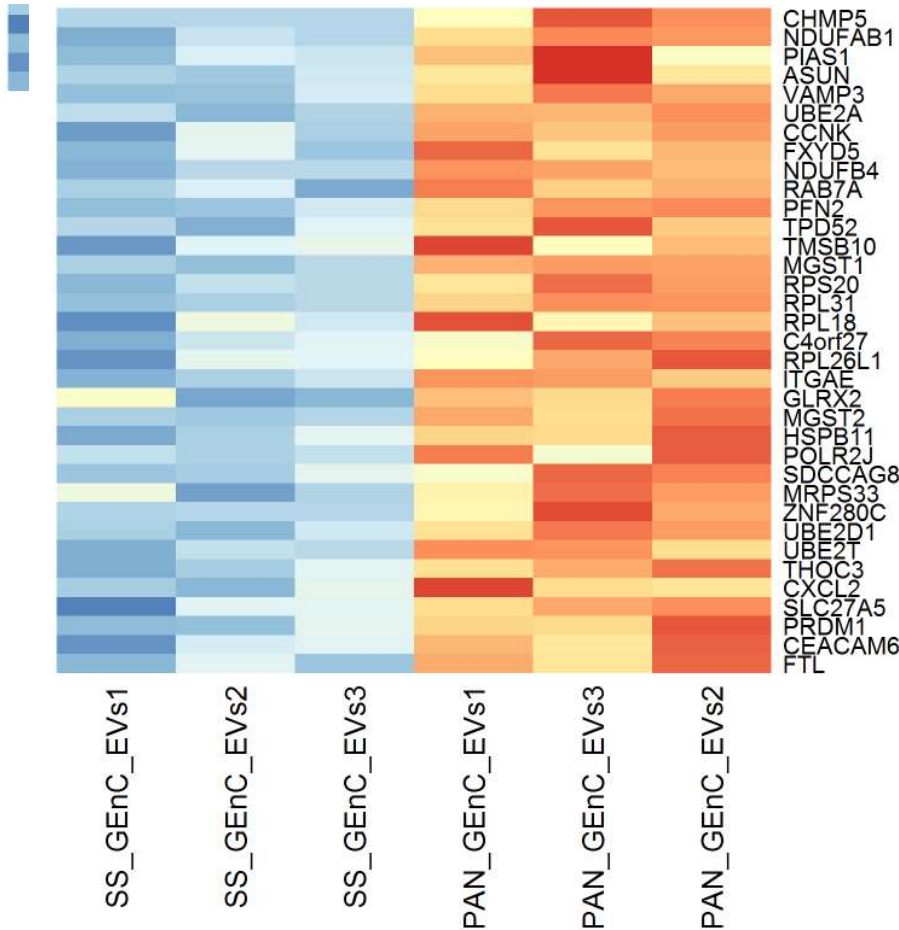

Supplement: S5 Fig — (PDF) [file pone.0224852.s005.pdf]

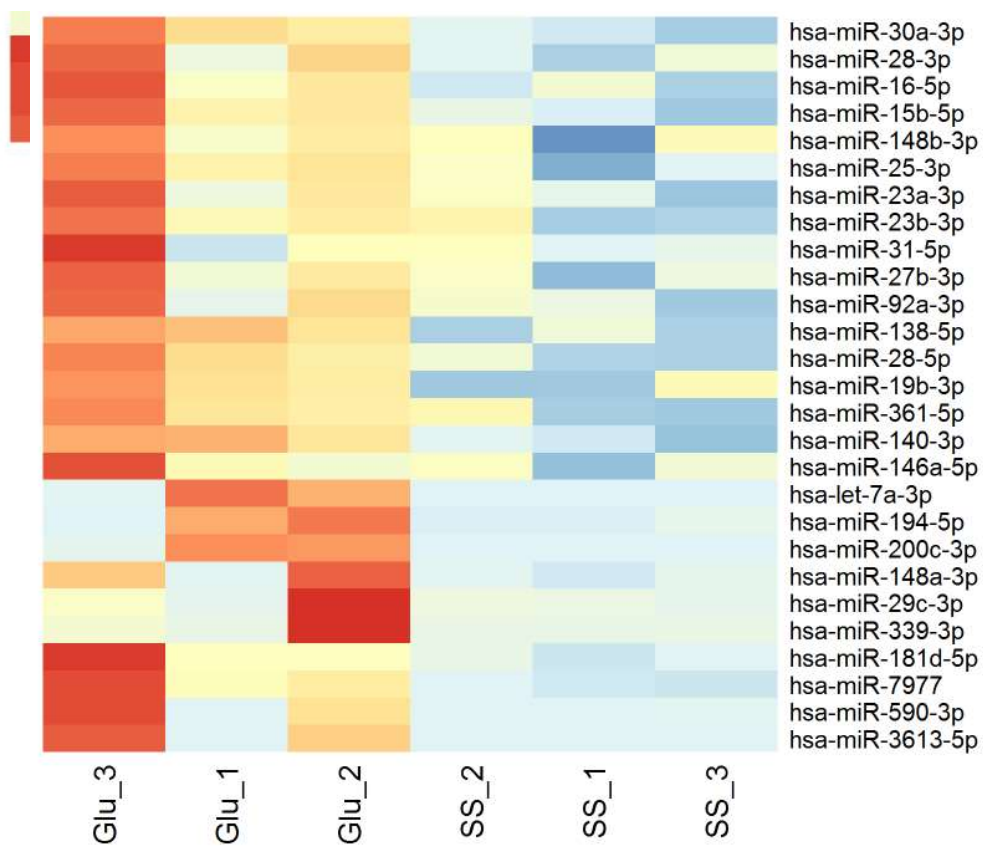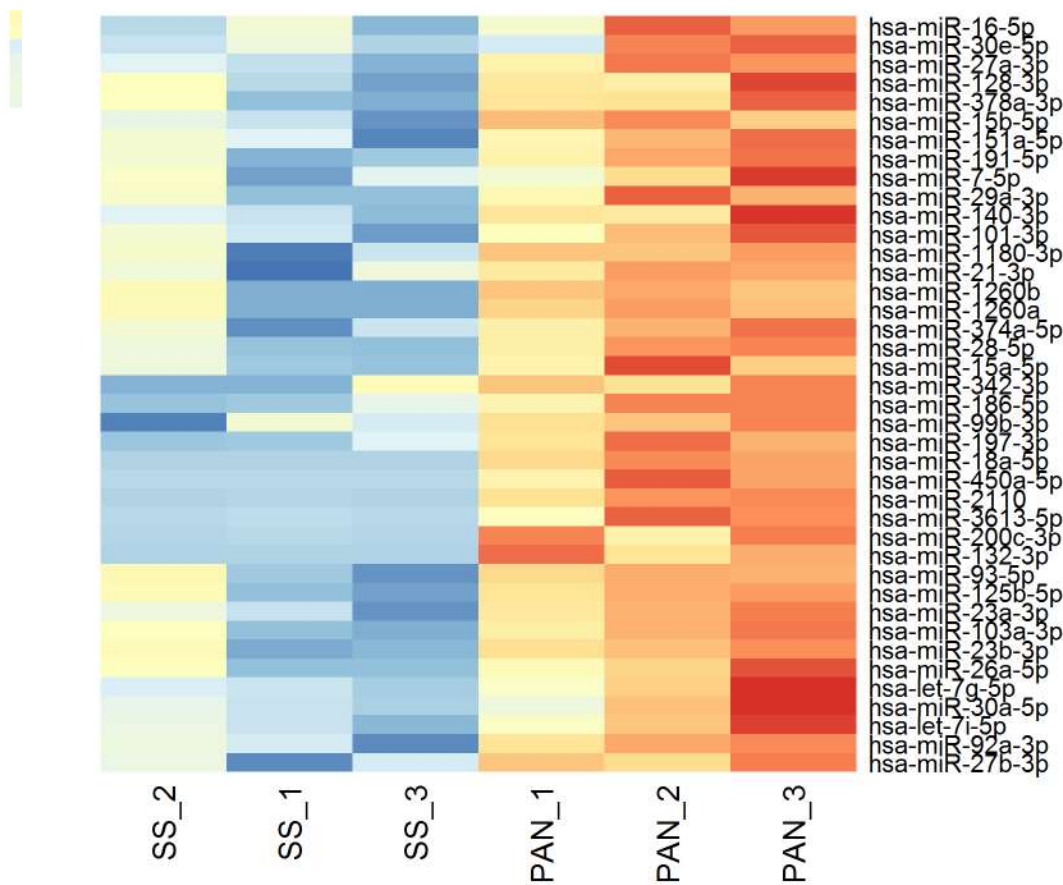

Supplement: S6 Fig — SS vs GLU (top 27) and SS vs PAN (top 40) comparisons for GEnC EV treated podocytes. (PDF) [file pone.0224852.s006.pdf]

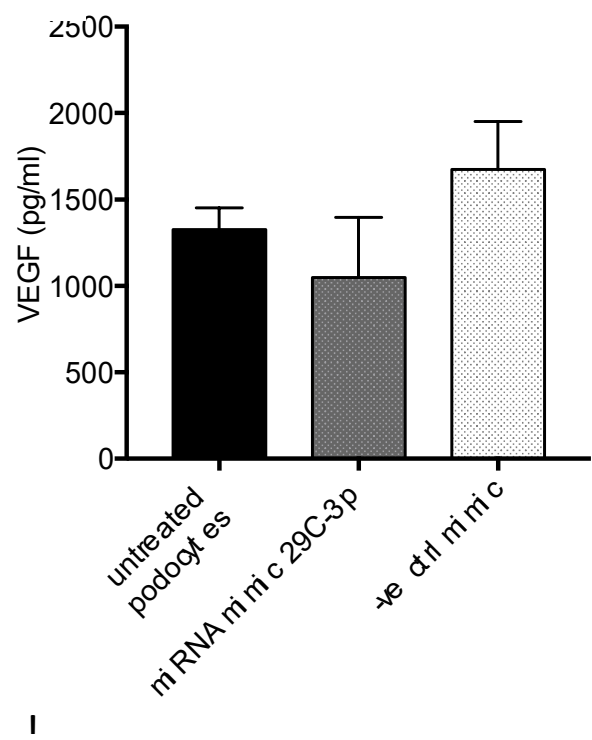

Supplement: S7 Fig — Negative control mimic was used as a control. n = 8. (PDF) [file pone.0224852.s007.pdf]
