# Supplementary material for: Glomerular endothelial derived vesicles mediate podocyte dysfunction: A potential role for miRNA
Source: PLoS One. 2020 Mar 26;15(3):e0224852. doi: 10.1371/journal.pone.0224852 (PMC7098579; doi:10.1371/journal.pone.0224852)
Supplement: S1 Methods — (PDF) [file pone.0224852.s008.pdf]

Extended materials and methods. Hill et al.

Human glomerular endothelial cells

Conditionally immortalised glomerular endothelial cells (GEnc) were a kind gift from Prof. M.Saleem to Imperial College Renal Unit. Cells were maintained and proliferated in a 33°C, 5% CO2, 95% humidified incubator. They were induced to maturity in a 37°C, 5% CO2, 95% humidified incubator. Cells were maintained at 37°C for 3-6 days before use. Culture flasks were coated with Attachment Factor (Thermofisher). Cells were cultured in Microvascular Cell Growth Kit media (ATCC). Media were renewed every 2-3 days and cells were subcultured at 60-70% confluency at a 1:3 cell density. Cells were used in experiments up to passage 41, as cells retain morphological features of GEnC up to this passage (1).

Human podocytes.

Due to cell availability, cost and relative ease of use, conditionally immortalised human podocytes were used in FM1-43X transfer and *C.elegans* miRNA mimetic transfer experiments. The conditionally immortalised human podocytes were a kind gift from Prof. M.Saleem to Imperial College Renal Unit (2). Cells were maintained and proliferated in a 33°C, 5% CO2, 95% humidified incubator. They were induced to differentiate in a 37°C, 5% CO2, 95% humidified incubator. Cells were maintained at 37oC for 14 days prior to use. Immortalised podocytes were grown and maintained in RPMI +10% PS + 25% FCS + 5% L-glutamine + 5% ITS. Media were renewed every 2-3 days and cells were split when 60-70% confluent.

Primary human podocytes were used for all other studies. Human podocyte primary cells were purchased from Celprogen and cultured following manufacturer's instructions. Primary podocytes were maintained and proliferated in a 37°C, 5% CO2, 95% humidified incubator. Podocytes were seeded onto Human Podocyte Primary Cell Culture Extra-Cellular Expansion Matrix precoated flasks (Celprogen). Podocytes were grown and maintained in Human Podocyte Primary Cell Culture Complete Media with Serum (Celprogen). Media were renewed every 2-3 days and cells were subcultured at 60-70% confluency, at a 1:2 cell density. Cells were used in experiments up to passage 12, as cells retain morphological features of podocytes at least up to this passage(3).

FM1-43 staining

GEnC were harvested and resuspended in 5µg FM1-43FX (Thermo Fisher Scientific) and incubated in the dark for 5 minutes. GEnC were washed to remove any remaining (free-floating) dye. GEnC were seeded onto transwell inserts for co-culture. In some experiments, cells were simulated with 1µg/ml LPS, as described above. Podocytes were collected, and backdrop suppressor reagent (Thermo Fisher Scientific) was added to quench any extracellular dye. FM1-43FX positive cells were counted by flow cytometry (BD AccuriC6) or imaged on a Leica SP5 inverted confocal microscope to detect FM1-43FX expression.

***C.elegans* miRNA mimetic transfection**

GEnC were transfected with Lipofectamine 2000 (Invitrogen) and a *Caenorhabditis elegans* (*C.elegans*) miR-39 miRNA mimetic (QIAGEN) according to manufacturer's instructions. Cells were incubated in a 37°C, 5% CO2, 95% humidified incubator for

24 hours, before GEnC-podocyte transwell co-culture. RNAseA (Invitrogen) was added to remove any remaining extracellular miR-39 mimetic. Post 24-hour culture RNaseA was re-added to remove any potential extracellular RNA. Total RNA was isolated from the cells using a miRNeasy kit, as per instructions (QIAGEN). Real time PCR (qRT- PCR) was performed following the QIAGEN protocol. Sequence of *C.elegans* miR-39: 5'-UCACCGGGUGUAAAUCAAAUCAGCUUG-3'.

RNA isolation and quantification
Total RNA was extracted from the cells using a miRNeasy kit (QIAGEN) and quantified using a NanoDrop 2000 spectrophotometer (Thermo Scientific). For RNA sequencing, RNA was purified from podocytes using the Total RNA Purification Kit (Norgen Biotec Corp) (see below).

BCA protein assay
EVs were lysed in NP40 + PI. A set of protein standards were prepared from a 2mg/ml albumin standard (BSA) ampule. The 2 set of dilutions ranging from 2000μg/ml to 25μg/ml. Reagent A and Reagent B were mixed at a 50:1 ratio to prepare a WR. A sufficient volume to pipette each sample in duplicates plus standards plus unknown. For the microplate procedure, 25μl of each standard or unknown sample plus 200μl of WR were added. The plate was incubated at 37°C for 30 minutes and absorbance was read at 650nm on a plate reader. The average absorbance of the blank standard was subtracted from the samples and unknown replicates. A standard curve was created to determine the protein concentration of EVs in μg/ml.

Western blot
EVs and cells were lysed in NP40 supplemented with protease inhibitors (Sigma). 1/5 β- mercaptoethanol was added to the loading buffer as a reducing agent. Samples were incubated at 95oC for 3 minutes and then run on 15% SDS-PAGE (sodium dodecyl sulfate polyacrylamide gel electrophoresis) (Invitrogen) at 100 volts, until the dye had reached the bottom of the gel. Proteins were then transferred to a nitrocellulose membrane (GE healthcare). Membranes were blocked in 10% milk for at least 30 minutes, washed 3x in TBST and incubated overnight in primary antibody. Membranes were then washed 3x in TBST. Protein was detected with horseradish peroxidase-conjugated secondary antibodies in combination with Amersham ECL Western Blotting Reagents (GE Healthcare).

Primary antibodies		
**Antigen**	**Clone**	**Manufacturer**
CD63	Polyclonal rabbit IgG	Abcam
GAPDH	Polyclonal goat IgG	R&D Systems
GRP94	ERP3988	Abcam
TSG101	EPR71130(B)	Abcam
Secondary antibodies		
HRP conjugated anti-goat	Polyclonal rabbit IgG	R&D Systems
HRP conjugated anti-rabbit	Polyclonal goat IgG	R&D Systems

RNA sequencing and analysis

RNA was sequenced using the Ovation Human FFPE RNA-seq Library Systems kit. The step- by-step protocol was as follows: First Strand cDNA Synthesis Using DNase-Treated, Second strand cDNA synthesis, cDNA fragmentation, cDNA purification, End Repair, Ligation, Strand selection, Strand Selection Purification, Strand Selection II, Adapter cleavage, Library amplification, Bead purification of amplified material, Quantitative and Qualitative assessment of Library. The size distribution and integrity of the library was checked using the Agilent 2100 BioAnalyzer. 1µl of cDNA was loaded into the highly sensitive DNA 1000 assay chip to determine results. cDNA was quantified using a Qubit Fluorometer; 199ul of Qubit Buffer was mixed with 1µl Qubit Reagent to make the reagent and 1µl of cDNA was added to the reagent and mixed thoroughly for absorbance reading. The concentration was determined as ng/ml. Equal concentrations of all individual libraries were pooled for multipex sequencing runs and concentrated using DNA Clean and Concentrator 5 kit (Zymo). Libraries were run independently on 3 individual sequencing lanes. Single end sequencing of multiplexed libraries was performed on an Illumina NextSEQ 500 at the Vanderbilt Technologies for Advanced Genomics (VANTAGE) core library (Vanderbilt University, TN, USA). The raw (not normalised) mRNA counts of 3 separate experiments for RNA sequencing reads were loaded into DESeq2, these counts were then normalised by variance-stabilising transformation. Data was organised with a cut off of minimum P value of 0.05 and fold change of >1.5.

Gene enrichment analysis was used to determine the biological function of the upregulated mRNAs. Using GO, (http://geneontology.org/page/go-enrichment-analysis) an online resource for analysis, data enrichment analysis was performed on the sets of genes that were significantly upregulated in the treated podocytes. This determined which gene ontology terms were over represented in the treated podocytes. PANTHER groups are annotated subfamilies of related genes that are likely to share function. The core module is a large protein library that contains all protein coding genes from 82 organisms, organised first into subfamilies on the basis of their shared functions. The results obtained from the analysis are therefore based on enrichment relative to the set of all protein coding genes in the human genome. The statistical overrepresentation test compares the uploaded gene list and it determines whether a particular class of genes is overrepresented. For each function category e.g. cell proliferation for biological processes, the binomial test is applied (from the PANTHER reference genome data set) to determine whether there is a statistical overrepresentation or underrepresentation of the genes in the test list relative to the reference list. http://bioinformatics.psb.ugent.be/webtools/Venn/ was used to create Venn diagrams. Statistically upregulated genes were inputted into the site to create Venn diagrams.

Small RNA sequencing and analysis

NEXTflex Small RNA Library Preparation Kits v3 for Illumina Platforms (BioScientific), was used to prepare small RNA libraries from total RNA as follows: NEXTflex 3'4N Adenylated Adapter Ligation, Excess 3' Adapter Removal, Excess Adapter Inactivation, NEXTflex 5'N Adapter Ligation, Reverse Transcription-First Strand Synthesis, Bead Cleanup, PCR amplification. Pippin

Prep was then implemented. 3% Agarose Gel Cassettes were used to collect fragments of 100-250bp. The cassette was prepared for loading as follows. Buffer was removed from the elution modules and replaced with 41µl of fresh electrophoresis buffer. The wells were sealed with an adhesive tape strip and continuity tested to calibrate the cassette. 50µl of buffer was removed from each sample well and 40µl of sample (30µl of sample plus 10µl of marker plus 5µl buffer) was loaded into the cassette. The library size distribution and integrity and concentration were assessed using the Agilent 2100 BioAnalyzer and Qubit fluorimeter, as previously described. Equal concentrations of all individual libraries were pooled for multiplex sequencing runs and concentrated using DNA Clean and Concentrator 5 kit (Zymo). Libraries were run independently on 3 individual sequencing lanes. Single end sequencing of multiplexed libraries was performed on an Illumina NextSEQ 500 as above. The raw (not normalised) mRNA counts of 3 separate experiments for RNA sequencing reads were loaded into DESeq2, these counts were then normalised by variance-stabilising transformation.

Data was organised with a cut off of minimum P value of 0.05 and fold change of >1.5. The miRNA enrichment analysis and annotation (MIEAA) tool (https://ccb-compute2.cs.uni- saarland.de/mieaa_tool/) was used as an overrepresentation analysis predictor. MIEAA calculates the significance of categories for a test set and determines if the specific category is over-represented or under-represented for the test set with respect to a reference set. Over 14,000 categories are available for analysis on the site. From the input data, a results table is created containing the category, subcategory, P-value (computed by applying the fishers exact test), expected and observed number of miRNAs, and the respective miRNAs per subcategory. http://bioinformatics.psb.ugent.be/webtools/Venn/ was used to create Venn diagrams. Statistically upregulated genes were inputted into the site to create Venn diagrams.

Seahorse Assays

Seahorse XFe96 analyser (Agilent) was used to measure oxygen consumption rate (OCR) and extracellular acidification rate (ECAR). We used the Seahorse XF cell Mitochondrial Stress test to measure mitochondrial respiration in cells by injecting concentrations of oligomycin (1µM), FCCP (2µM), and rotenone/antimycin A (0.5µM), into the cell media according to manufactures instructions. We also used the Seahorse XF cell Glycolysis Stress test to measure glycolysis in cells by injecting concentrations of glucose (100mM), oligomycin (10µM), and 2-deoxy-glucose (500mM) into the cell media according to manufactures instructions. Briefly, primary human podocytes were harvested from a T75 flask, counted and subsequently seeded onto a Seahorse 96-well XF cell culture plate at 4×10^4 cells/well. Podocytes were left for 12 hours to adhere to the plate. Cells were excluded from the top row of the XF96 well plate as they served as background wells. Podocytes were left untreated or EVs derived from steady state, glucose, LPS or PAN treated GEnCs were added to the podocytes and cultured for 24 hours in Seahorse XF Media. The Seahorse XF96 sensor cartridge was hydrated in 200µl of Seahorse XF calibrant solution. The cartridge was left in a in a CO_2-free, 37°C incubator overnight before podocyte measurements. Measurement started after calibration and 2 min of pre-mixing.

After drug exposure 12 OCR and ECAR measurements are made every 6 min for at least 12 measurements. Changes in respiration are analysed according to manufactureres instractions (Agilent).

Mitochondrial staining

Podocytes were treated with GEnC EVs at a 4:1 cell ratio or left untreated for 24 hours. Coverslips with adhered podocytes were washed 2x in PBS, and mitochondria was stained using 100nm Mitotracker Deep Red FM (Life technologies). Cells were washed and fixed in 4% PFA for 15 minutes. Cells were mounted in DAPI-vectashield (Vector labs) and imaged using a Leica SP5 confocal microscope.

ROS staining

MitoSOX Red reagent is a mitochondrial superoxide indicator fluorogenic dye. MitoSOX produces red fluorescence when oxidised by superoxide. Podocytes were treated and harvested using cell dissociation buffer (Thermo Fisher Scientific) and stained with 5µm MitoSOX Red (Thermo Fisher Scientific). 10 minutes after staining, cells were analysed by flow cytometry on a BD LSRFortessa X-20.

VEGF measurement.

Concentrations of VEGFA, were measured using Duoset ELISA kits (R&D systems, Abingdon, UK), according to the manufacturers protocol. Absorbance was measured at 450nm using a Bio-Tek ELX 800 microplate reader (Bio-Tek Instruments).

Podocyte actin staining

Coverslips with adhered, EV or control treated podocytes were washed 2x in PBS, fixed in 4% PFA for 15 minutes and washed with 0.1M glycine. Cells were permeabilised with Triton X-100 and actin was stained using fluorescently conjugated phalloidin (Life technologies). Cells were mounted in DAPI-vectashield (Vector labs) and imaged using a Leica SP5 confocal microscope.

miRNA mimic transfection

$1x10^5$ podocytes in 24 well plates were transfected with Lipofectamine 2000 (Invitrogen) and miRNA mimic miR-200C-3p or miR-29C-3p or negative control miRNA mimic (Life technologies) according to manufacturer's instructions. Cells were incubated for 24 hours. Supernatant was then removed, and cells were washed. Fresh media was then added to the podocytes and supernatant was collected 24-hours later. VEGF ELISA was performed as described above.

1. Satchell SC, Tasman CH, Singh A, Ni L, Geelen J, von Ruhland CJ, et al. Conditionally immortalized human glomerular endothelial cells expressing fenestrations in response to VEGF. *Kidney Int.* 2006;69(9):1633-40.
2. Saleem MA, Ni L, Witherden I, Tryggvason K, Ruotsalainen V, Mundel P, et al. Co-localization of nephrin, podocin, and the actin cytoskeleton:

evidence for a role in podocyte foot process formation. *Am J Pathol.* 2002;161(4):1459-66.

3. Harvey TW, Engel JE, and Chade AR. Vascular Endothelial Growth Factor and Podocyte Protection in Chronic Hypoxia: Effects of Endothelin-A Receptor Antagonism. *Am J Nephrol.* 2016;43(2):74-84.
