# Supplementary material for: Glomerular endothelial derived vesicles mediate podocyte dysfunction: A potential role for miRNA
Source: PLoS One. 2020 Mar 26;15(3):e0224852. doi: 10.1371/journal.pone.0224852 (PMC7098579; doi:10.1371/journal.pone.0224852)
Supplement: S1 Data — (PPTX) [file pone.0224852.s009.pptx]

## Slide 1
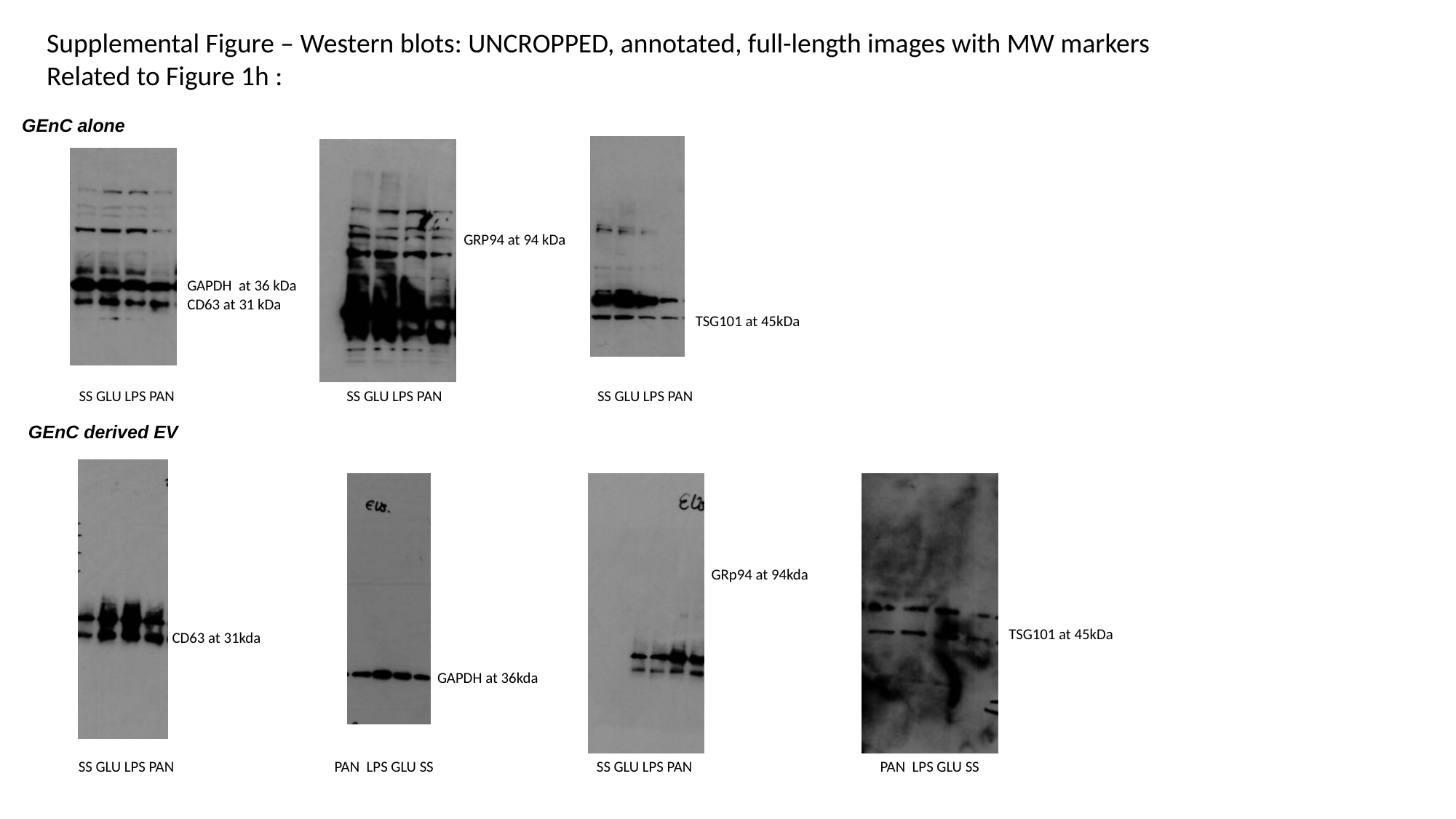

Supplemental Figure – Western blots: UNCROPPED, annotated, full-length images with MW markers
Related to Figure 1h :
GEnC alone
GRP94 at 94 kDa
GAPDH at 36 kDa
CD63 at 31 kDa
TSG101 at 45kDa
SS GLU LPS PAN
SS GLU LPS PAN
SS GLU LPS PAN
GEnC derived EV
GRp94 at 94kda
TSG101 at 45kDa
CD63 at 31kda
GAPDH at 36kda
SS GLU LPS PAN
PAN LPS GLU SS
SS GLU LPS PAN
PAN LPS GLU SS
