# Supplementary material for: Glomerular endothelial derived vesicles mediate podocyte dysfunction: A potential role for miRNA
Source: PLoS One. 2020 Mar 26;15(3):e0224852. doi: 10.1371/journal.pone.0224852 (PMC7098579; doi:10.1371/journal.pone.0224852)
Supplement: Attachment — Submitted filename: rebuttal PLOSone.docx [file pone.0224852.s010.docx]

Thank you to editorial board and reviewers for their time taken to review our paper. We have now addressed all editorial concerns and journal requirements. We have added point-by-point rebuttal to reviewers comments below.

Reviewer #1: Comments:

The authors inquire about the role played by extracellular vesicles (EV) trafficking between two different types of cells in the progression of kidney disease. To this aim, they use a non-contact in vitro transwell system to characterise glomerular endothelial cell to podocyte communication. Using this experimental setting, they try to get more insights into how extracellular signals from the glomerular microenvironment regulate podocyte activity.


This manuscript describes carefully performed in vitro work which demonstrates that upon injury, EVs are secreted by glomerular endothelial cells and up taken by adjacent podocytes. This mechanism leads to alteration in podocyte mRNA and miRNA content and subsequent changes to podocyte functions.
The experimental design is well set up and the conclusions are presented in an appropriate fashion and are supported by the data.

Thank you for the time taken to review our paper and for the encouraging comments in your review. We have added a rebuttal to each minor issue below.


Minor issues:

1) However, there is no in vivo relevance which support these findings and no evidence this extracellular transfer is taking place in the injured glomerulus. By quoting previous work (ref 41), the authors argue their described extracellular trafficking can occur in vivo since EVs, intravenously injected in the mouse, were found in the urine, implying active in vivo transfer through the glomerular basal membrane.
In the present work, the authors demonstrated they can efficiently produce and isolate EVs from quiescent and injured glomerular endothelial cells, i.e. 3.1010 EVs per 1.106 cells. They also demonstrated uptake of EVs by podocytes using the fluorescent membrane probe FM1-43X. One can argue they could have produce high levels of fluorescently-labelled EVs, systemically injected them in the mouse and check for FM1-43X staining in podocytes.

Thank you for your comments. We agree that we do not show direct in-vivo relevance. Our work establishes an in-vitro based method to show endothelial and podocyte transfer of endothelial vesicles. We are now applying for grants to complete work to show direct in-vivo efficacy, but this is out of scope for the current manuscript.

2) In Figure 2 panels D and E, glomerular endothelial cells were transfected with C.elegans Cel-mir-39 mimetic and further transwell cultured with podocytes. Podocyte incorporated the miRNA which proved successful exogenous transfer.
However, Panel E shows that Cel-mir-39 was significantly increased in the LPS-treated condition. It is not clear how that happened since Figure 1 showed that the number of EVs was not affected by endothelial cell activation (LPS, Glucose and PAN). Although it is clear that the EV content changes from quiescent to activated endothelial cells as it is able to alter podocyte mRNA and miRNA, why would it this affect Cel-mir-39 abundance?

Thank you for this insightful comment. We were also intrigued by the observation that LPS treated endothelial cells transferred more Cel-mir-39 even though, as rightly pointed out, LPS did not seem to increase total number of glomerular endothelial secreted EVs or transfer (as shown in by FM1-43, Fig2C). We do not have a clear answer as to why LPS would affect Cel-mir-39 abundance. Our current working hypothesis is that LPS may increases some miRNA EV loading and/or transfer of miRNA transfer and EV from endothelial cell donors to podocyte recipients. These are difficult experiments to design with conclusive results and as such is work in progress and outside the scope of the current manuscript.

3) There is no error bars in Figure 5C although the analysis seems to have been performed in triplicate for each conditions (Supplemental Figure 6).

Apologise, this was an oversight on our part. Thank you for bringing this to our attention. Error bars have now been added to Figure 5C.

Reviewer #2: This manuscript shows in-vitro experiments describing the glomerular endothelial cells to podocyte communication via extracellular vesicles in a variety of conditions and focuses on miRNA transfer between these cells.
The manuscript is well written; the background is well described. Experimental methods are also well described. Many data are shown in order to confirm the hypothesis.

Thank you for the time taken to review our paper and for the encouraging comments on our manuscript, we have added a rebuttal to each minor comment below.
I have a few minor comments:
- On the one side, focus has been put on extracellular vesicles. In order to display all the aspects of cell-to-cell communications, the authors should also describe the other types of transfer (protein-boud miRNAs, apoptotic bodies). In this line, authors should specify if cel-miR-39 might be transferred by these mechanisms instead of EV. If their experiments cannot be conclusive they should add this limitation in their Discussion Section.

Thank you for your comment. We understand your concerns that we do not directly show EV transfer of cel-miR-39, but a body of evidence to show EV release and EV containing miRNA. Nevertheless, we have now made a comment that we cannot be conclusive of EV transfer of cel-miR-39 in discussion section (pg17, second paragraph).

- On the other side, focus has been put on one specific component of EV, miRNAs. However, to the best of my knowledge, EV also carry other component such as nucleic acids, proteins, and lipids, all of which might induce various cellular response in the recipient cells. Authors should discuss this point. In the same line, might EV carry the treatments that GenC received (glucose, PAN, LPS)? In this case the effects seen on recipient cells could also be due to treatment transfer, which is also interesting if possible. Please discuss this point with appropriate references.

Thank you for your comments. We understand and agree that other components of EVs may also induce cellular responses in recipient cells or potential treatment transfer. We have now added comments to this in discussion with appropriate references. (pg17, last paragraph).

- Is the chosen pore size of the transwell system appropriate to reflect glomerular filtration barrier? Please justify this in the Methods Section.

Poor size was chosen to prevent direct cell migration and direct endothelial-podocyte contact. (pg6, 2^nd^ paragraph).

- Results regarding the role of miRNA-200c-3p are not sufficient in my opinion in order to establish a causal relationship between EV transfer of miRNA-200c-3p and VEGF production. First, the amount of miRNA-200c-3p transferred by EV on the one side, and by transfection on the other side, is not specified. The amounts are likely to be very different. Second, other components of EV might induce the decrease in podocyte VEGF production. Finally, podocyte impairment by either mechanism might result in VEGF decrease, rather than being the consequence of VEGF decrease. For these reasons, I suggest to authors to discuss this point, and to remove “via extracellular transfer of miRNA-200c-3p” from the title of the article, which is too assertive compared to the actual results of the experiments. The title might be “glomerular endothelial derived vesicles mediate podocyte dysfunction: a potential role for miRNAs” for example.

We understand your concerns and have changed title to “Glomerular endothelial derived vesicles mediate podocyte dysfunction: a potential role for miRNA”
